# Combined Subacromial Bursal Stem Cell Therapy and Platelet-Rich Plasma Alongside Arthroscopic Rotator Cuff Surgery Reduces Postoperative Pain and Improves Functional Outcomes: A Retrospective Study

**DOI:** 10.3390/jcm14155590

**Published:** 2025-08-07

**Authors:** Mladen Miškulin, Josip Savić, Oliver Dulić, Emili Dragaš, Andro Košec

**Affiliations:** 1 Special Hospital Arithera, 10000 Zagreb, Croatia; miskulinmladen@gmail.com (M.M.); dr.med.josip.savic@gmail.com (J.S.); 2 Clinical Center of Vojvodina, Medical Faculty, University of Novi Sad, 21000 Novi Sad, Serbia; oliver.dulic@mf.uns.ac.rs; 3 School of Medicine, University of Zagreb, 10000 Zagreb, Croatia; emili.dragas1@gmail.com; 4 Department of Otorhinolaryngology and Head & Neck Surgery, University Hospital Center Sestre Milosrdnice, 10000 Zagreb, Croatia

**Keywords:** stem cell therapy, shoulder pain, surgery, rotator cuff injury

## Abstract

**Background/Objectives**: This study investigates the benefits of incorporating stem cell therapy into arthroscopic rotator cuff repair by evaluating its impact on postoperative pain and functional recovery. **Methods**: A retrospective, comparative analysis was conducted with a small cohort of patients undergoing rotator cuff surgery, divided into two groups: one receiving adjunctive combined PRP and bursal stem cell therapy and the other undergoing standard arthroscopic repair alone. The outcomes were assessed using visual analog scale (VAS) scores for pain and the Constant–Murley score (CMS), which includes strength of abduction, VAS pain, limitation and range of motion, evaluated at baseline, 1, 2, 3 and 6 months postoperatively. **Results**: Patients in the stem cell group experienced significantly greater reductions in pain scores and more substantial improvements in functional scores at the follow-up points compared to the control group. A linear mixed-effects analysis showed that in the early postoperative period, the use of PRP and bursal stem cell therapy was associated with significantly reduced postoperative VAS pain scores (F 4.8, *p* = 0.045) and an increased CMS regarding postoperative pain (F 8.6, *p* = 0.01), alongside painless elevation level (F 6.5, *p* = 0.022), forward flexion (F 8.5, *p* = 0.01) and abduction scores (F 8.3, *p* = 0.011). The effect of PRP and bursal stem cell therapy remains constant during late follow-up, from the fourth to sixth postoperative month, with postoperative CMS regarding pain remaining statistically significantly higher in the stem cell therapy group (F 4.8, *p* = 0.008), alongside reduced night-time pain (F 7.4, *p* = 0.015), improved recreation ability (F 4.8, *p* = 0.044) and reduced activity restriction (F 5.8, *p* = 0.028). **Conclusions**: The findings suggest that the addition of stem cell therapy to arthroscopic rotator cuff repair may enhance postoperative recovery by alleviating pain and promoting functional gains.

## 1. Introduction

Shoulder pain is a common complaint in many patients that may require surgery, and it most often occurs due to the pathology of the rotator cuff [1]. Rotator cuff tears (RCTs) occur in both athletes and the general population, significantly increasing with age, following repeated overhead movements of the upper arm and subsequent repeated and chronic tendon injuries. Without proper treatment, they lead to rotator cuff arthropathy following glenohumeral arthritis and consequent pain, a decrease in range of motion (ROM) and disability [2]. This can lead to an inability to work in the working population and a loss of functional independence in older adults, creating a serious functional deficit in everyday life [3]. The most common sites of injuries are the supraspinatus and the subscapularis muscles and their tendons, which are responsible for most of the abduction and internal rotation of the shoulder joint [4]. The treatment of RCTs includes both surgical and non-surgical options. When determining the right treatment, persistent pain, loss of ROM and strength, patient age, time elapsed from injury and factors regarding anesthesia are taken into consideration. Conservative treatment, including physiotherapy and/or cortisone injections, is a starting point and preferred in patients with fewer physical demands and in older adults [2]. Surgery is often used in treatment, and, over time, surgical techniques have become less invasive, with arthroscopy now being the gold standard, as opposed to an open approach, leading to a shorter patient recovery time [1,5]. Although there have been improvements in surgical techniques, the success rate is often lower than desired, with healing being a limiting factor in patient recovery. This is attributed to the hypovascularity of tendons, along with age-related degenerative changes. As a result, there has been growing interest in regenerative medicine, which seeks to leverage the body’s natural ability to repair and restore tissue function and structure [6,7]. Several biological augmentation strategies, including growth factors, platelet-rich plasma (PRP), mesenchymal stem cells (MSCs) and their combinations, have been studied [6]. MSCs are strongly associated with the acceleration of tissue regeneration due to their strong self-renewal ability and pluripotency, allowing them to differentiate into different tissue types of the mesoderm [4,8]. Consequently, researchers have sought to identify the optimal source for MSC derivation. The subacromial bursa, situated between the rotator cuff and the acromion, has been observed to regenerate itself following surgical resection, highlighting its strong regenerative potential [7]. An in vitro study by Morikawa and colleagues showed that subacromial bursal stem cells have higher differentiation ability in comparison with bone marrow stem cells (BMSCs) extracted from the proximal humerus [6]. The favoring of subacromial bursal MSCs over BMSCs due to their differentiation abilities has been shown in numerous other studies [4,9]. Currently, there are a lot of in vitro studies indicating high potential for the use of stem cell therapy in surgery of the shoulder joint, given the well-established healing potential of stem cells.

Our goal was to explore the impact of using stem cell therapy alongside arthroscopic rotator cuff surgery on reducing postoperative pain and improving functional outcomes. To achieve this, we compared a group of patients who received stem cell therapy alongside arthroscopic surgery with a group that underwent surgery alone.

## 2. Materials and Methods

This is a single-center cohort retrospective comparative study on patients undergoing arthroscopic shoulder surgery, whose data were obtained in the period from 2021 to 2024, assembled according to STROBE guidelines. Written informed consent was obtained from all of the eligible patients. The study was conducted in accordance with the Declaration of Helsinki and approved by the Special Hospital Arithera Institutional Review Board on 30 April 2025.

**Scheme 1:**
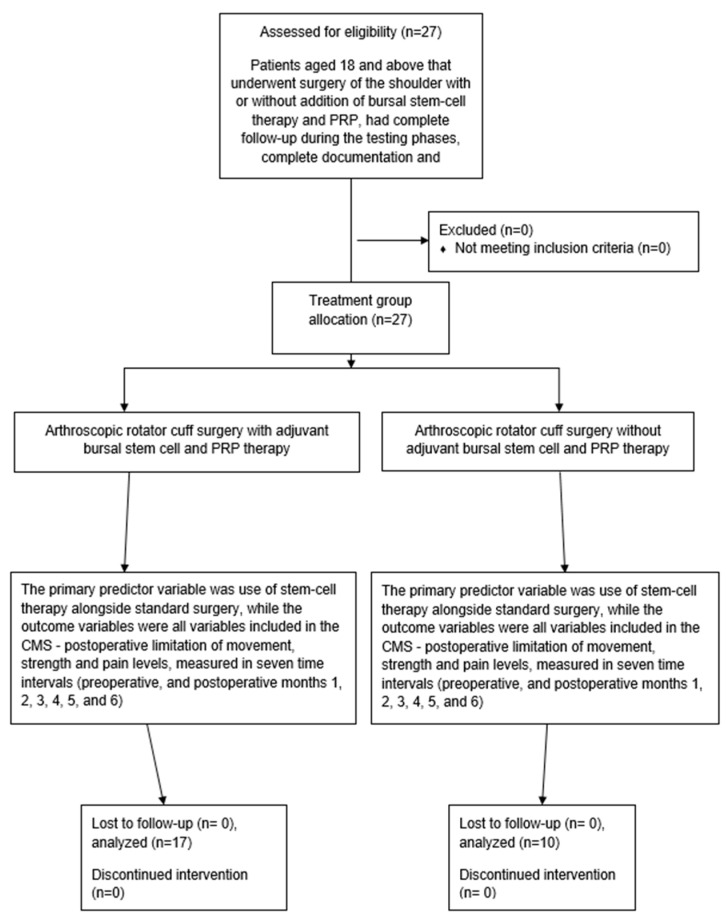
STROBE flowchart of the study protocol.

Inclusion criteria were patients aged 18 and above that underwent surgery of the shoulder with or without the addition of stem cell therapy and PRP, completed follow-up during the testing phases and completed the documentation and questionnaires (Scheme 1).

Indications for arthroscopic surgery included debilitating functional restriction due to supraspinatus tendon injury resulting in pain, weakness in abduction and functional limitations in everyday life, with severely limited active and passive external rotation (<30° at 0° abduction), and previous failed conservative management (including physiotherapy and/or cortisone injections or PRP injections). All surgeries were performed by a single senior, fellowship-trained shoulder surgeon. Patients were excluded if they had had previous surgery, were diagnosed with neurovascular disorders of the ipsilateral shoulder resulting in muscle weakness or loss of function, or had incomplete follow-up. Since this was a retrospective study, the group of patients not undergoing stem cell therapy was included before the therapy became routinely available, and the group of patients receiving stem cell therapy was included after a protocol for its use was established.

The covariates were age, sex, laterality, prior exercise, shoulder tendon, width of muscle rupture injury (size of the tear noted as small < 3 cm, medium > 3.5 cm but below 5 cm, and massive > 5 cm), Constant–Murley score (CMS), which includes strength of abduction in kilograms pre- and postoperatively, VAS pain, limitations (activity, recreation, night-time pain, arm elevation free of pain), and range of motion (forward flexion, abduction, external and internal rotation, and the combination of these movements, i.e., abduction–external rotation and adduction–internal rotation). The primary predictor variable was the use of stem cell therapy alongside standard surgery, while the outcome variables were all variables included in the CMS—the postoperative limitation of movement and strength and pain levels, measured at seven time intervals (preoperatively and at postoperative months 1, 2, 3, 4, 5, and 6). All patients underwent the same postoperative regime of physiotherapy and were scored by the same surgeon.

The postoperative rehabilitation protocol follows general recommendations for arthroscopic rotator cuff repair. The shoulder was protected with a shoulder sling in a position comprising adduction and slight internal rotation for four weeks. During this period, guided passive mobilization exercises were performed gradually for up to 90 degrees of abduction and 90 degrees of forward flexion, while extension was forbidden. Active rotation exercises were allowed through adduction, and the elbow and wrist were used in normal daily activities such as eating, drinking, hair brushing and working on a computer. The sling was discarded gradually in the fifth postoperative week.

Active assisted and active non-assisted ROM exercises started from the 5th postoperative week, aiming for full ROM, which is expected approximately after 10–12 weeks. Strengthening exercises were started along with the active ROM exercises, and the submaximal strength was expected to be achieved 4–6 months after surgery. A return to shoulder-demanding work or sports is usually possible after 6 months, depending on a patient-specific assessment.

A total of 27 patients without major comorbidities were included in this study, with 17 receiving stem cell therapy and 10 not receiving stem cell therapy.

The Constant–Murley score involves a 100-point scale composed of a number of individual parameters. These parameters define the patient’s level of pain and ability to carry out their normal daily activities. The CMS was introduced to determine the patient’s functionality after undergoing treatment for a shoulder injury. The test is divided into four subscales: pain (15 points), activities of daily living (20 points), strength (25 points) and range of motion, including forward elevation, external rotation, abduction and internal rotation of the shoulder (40 points). The higher the score, the higher the quality of the function.

The subjective findings (severity of pain, activities of daily living and working in different positions) for the participants contribute 35 points and objective measurements (ROM without pain, exo- and endo-rotation measurements via reference points and muscle strength) comprise the remaining 65 points [10].

### 2.1. Surgical Technique and Postoperative Protocol

**Figure 1:**
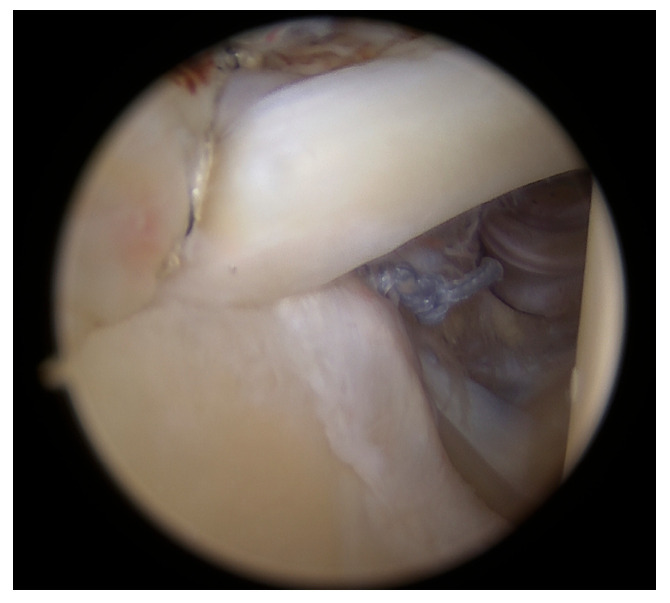
Positioning of the sutures.

All procedures were performed by the senior author with patients positioned in the beach chair position under general anesthesia. This position was preferred over the lateral decubitus position because it keeps the shoulder in its natural alignment, similarly to in everyday activities, and prevents excessive stretching of the surrounding structures. This allows tendons to be placed over the footprint under natural tension, reducing the risk of overstretching, which could lead to weakness in abduction or early re-tearing, particularly in cases with highly degenerated tendons. The subacromial space is no larger than in everyday activities, like it would be in traction devices, enabling us to standardize the amount of acromioplasty. At the end of treatment, during surgery, the arm is free to be moved in any direction necessary for good visualization of the tear, mostly through rotations. An additional venous line was placed in the contralateral arm to harvest venous blood free of drugs and infusion solutions. The procedure began with the establishment of a standard posterior portal, followed by a thorough standardized intra-articular inspection to confirm MRI and clinical findings. Common intra-articular pathologies, such as SLAP 2 tears or degenerative lesions of the long head of the biceps (LHB), with or without subscapularis tendon involvement, were addressed accordingly. The standard anterosuperior approach was used for debridement of the superior glenoid, tenotomy of the LHB and suture management for the subscapularis or LHB tendon, if necessary. A superolateral portal was carefully positioned to facilitate both SLAP repair and later supraspinatus reconstruction. The SLAP 2 lesion was first cleared of fibrous tissue, and the superior glenoid was debrided to the cortical bleeding bone using a motorized shaver. Through the superolateral portal, a double-loaded titanium CorkScrew Anchor 5.0 (Arthrex, Munich, Germany) was placed slightly posterior to the origin of the long head of the biceps, with sutures passed and tightened in a horse-saddle manner—one posterior and one anterior to the LHB origin (Figure 1)—while subscapularis repair and LHB tenodesis were performed via a direct anterior approach using a similar debridement and scarification protocol, securing the tendon with extra-articular knots.

**Figure 2:**
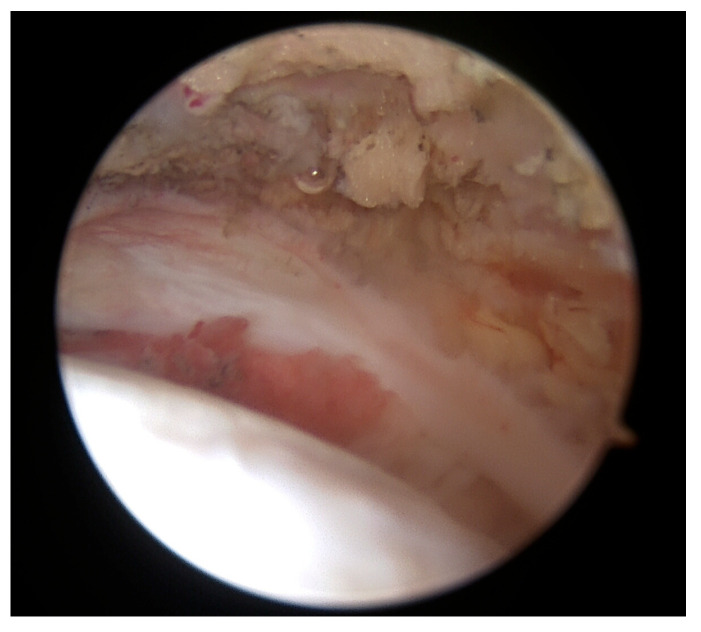
Harvesting subacromial bursal tissue.

Once the intra-articular procedures were completed, the arthroscope was repositioned into the posterior subacromial space view. A GraftNet device (Arthrex, Munich, Germany) was used to harvest subacromial bursal tissue, serving both as an augmentation material and to improve visualization and measurements of the tear (Figure 2).

**Figure 3:**
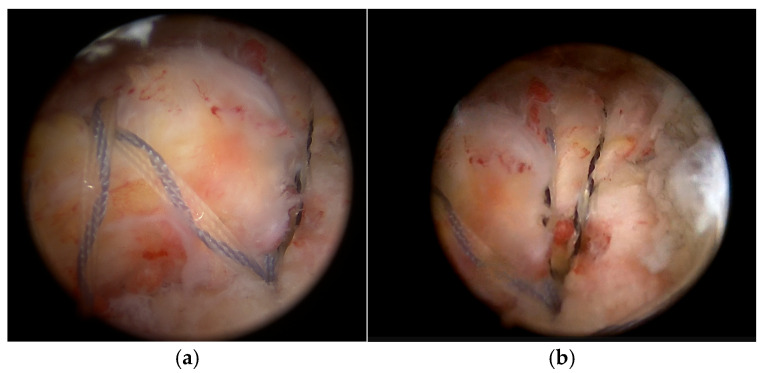
Double-row repair using titanium anchors (**a**) and a suture bridge fixation (**b**).

During the surgery, approximately at the time when enough bursal tissue had been collected, 15 mL of venous peripheral whole blood was drawn from one of two major veins from the contralateral arm, the median cubital or antebrachial part of the cephalic vein, using a 1 Arthrex ACP double syringe (Arthrex ACP, Munich, Germany). The blood was processed using a Hettich centrifuge at 1500 rpm for 5 min, obtaining 4–7 mL of autologous conditioned plasma (Arthrex ACP, Munich, Germany), which was then mixed by a scrub nurse with the harvested bursal tissue in a 1:0.3 ratio (bursal tissue:ACP) using 2 syringes connected through a female-to-female adapter. By pushing back and forth several times, a uniform pasty mass was created, and a 1 mL syringe was connected to the application cannula. Then, the fragments were transferred into the applied cannula and were carefully pushed up to the cannula tip using a trocar until they appeared at the opening. Meanwhile, the supraspinatus and infraspinatus footprints were prepared by removing fibrotic tissue and debriding down to the cortical bleeding bone to optimize healing conditions. A double-row repair technique was used, with the first row anchored near the cartilage border using titanium CorkScrew anchors (Arthrex, Munich, Germany), followed by the fixation of a suture bridge with BioPushLock anchors (Arthrex, Munich, Germany) (Figure 3a,b).

**Figure 4:**
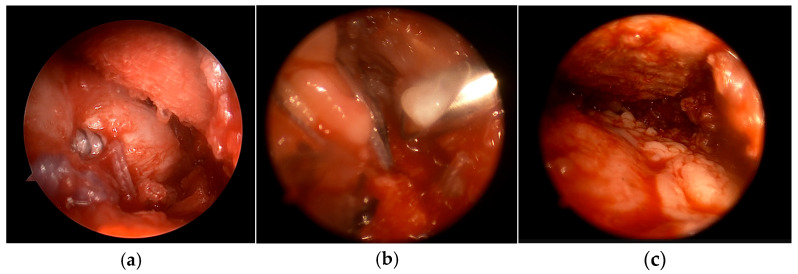
(**a**) Application of PRP–bursal tissue mixture all over the reconstruction site through one of the lateral portals (**b**) using the applicator under direct arthroscopic visualization (**c**) through the posterolateral portal.

To complete the procedure, acromioplasty was performed via lateral and posterior portals, with the extent of resection standardized using an acromizer shaver blade 5.0 (Arthrex, Munich, Germany). At the end of the reconstruction and acromioplasty, the reconstruction site was dried out in order to prevent migration of the augmenting tissue. Once the subacromial space was dried, the PRP–bursal tissue mixture was applied all over the reconstruction site (Figure 4a–c) through one of the lateral portals using the applicator under direct arthroscopic visualization through the posterolateral portal and the wounds were closed. The remaining PRP was injected intra-articularly before covering the incisions.

Postoperatively, the shoulder was immobilized in a sling for four weeks, allowing only a passive range of motion of up to 90 degrees through abduction and forward flexion. Gradual passive rotations were introduced, progressing to active assisted movement and active non-assisted movement from the fifth postoperative week onward.

### 2.2. Statistical Analysis

A sample power analysis was conducted based on the published values of the preoperative and postoperative CMS. Alpha was set at 0.05, and the power of the test was 80%. The power analysis set the minimum required sample size to establish statistical significance at 23 subjects in the patient group, with an average preoperative CMS of 44, a standard deviation of 5, and a cut-off value denoting a significant change of 5 points.

The data distribution was calculated using the Kolmogorov–Smirnov test. Statistical analysis was performed using the Mann–Whitney U test or Friedman test (the non-parametric alternative to the one-way ANOVA with repeated measures) for paired samples depending on the normality of the distribution, performed to assess the correlations between CMS scoring intervals. A linear mixed-effects model (LMM), adjusted for baseline factors, was used to model the group × time interaction in one step. The LMM was fitted with outcomes such as the VAS and subcomponents of the CMS as the dependent variables, including fixed effects for treatment group, time and their interaction. All tests of statistical significance were performed using a two-sided 5% type I error rate.

Statistical analysis was performed using MedCalc software (Version 11.2.1 © 1993–2010. MedCalc Software bvba Software, Broekstraat 52, 9030 Mariakerke, Belgium) and SPSS (Version 22.0., 2013. IBM SPSS Statistics for Windows, Armonk, NY, USA: IBM Corp.) using standard descriptive statistics and frequency tabulation as indicated.

## 3. Results

The average age of our patients was 57.04 years, and there were 12 male and 15 female subjects. To ensure that the two groups were comparable at baseline, a group analysis of demographic and clinical characteristics was performed, with no significant differences in baseline characteristics (chi-square test, *p* > 0.05, Table 1).

**Figure 5:**
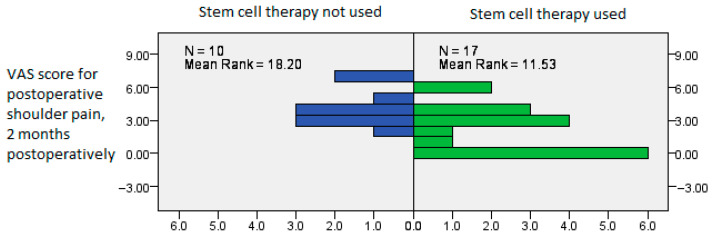
Postoperative VAS showing significantly improved results in patients treated with stem cell therapy, measured in the second postoperative month interval (Mann–Whitney U test, *p* = 0.031).

**Figure 6:**
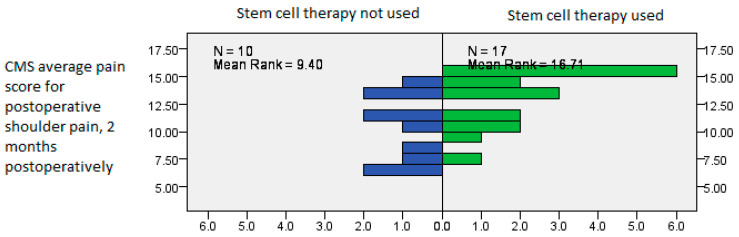
Postoperative CMS average scores showing significantly improved results in patients treated with stem cell therapy, measured in the second postoperative month interval (Mann–Whitney U test, *p* = 0.020).

We performed Mann–Whitney’s U test to see whether any of the covariates were associated with the predictor variable, stem cell therapy, with the postoperative VAS and pain-related CMS measured in the second postoperative month interval showing a significantly improved result in patients treated with stem cell therapy (Mann–Whitney U test, *p* = 0.031, *p* = 0.020, Figure 5 and Figure 6).

We then performed a linear mixed-effects analysis with subcomponent CMSs as the dependent variables. When analyzing range of motion variables, we tested preoperative pain, limitation of movement in activity and recreation, night-time pain, pain-free elevation, forward flexion, abduction, external rotation, internal rotation, range of motion scores and strength. The measurements were repeated at every check-up interval, and the analysis showed significant correlations between the treatment type and the range of motion subsections of the CMS.

The results in the early postoperative period showed that the use of PRP and bursal stem cell therapy was associated with significantly reduced postoperative VAS pain scores (F 4.8, *p* = 0.045) and an increased CMS regarding postoperative pain (F 8.6, *p* = 0.01), alongside significantly increased painless elevation level (F 6.5, *p* = 0.022), forward flexion (F 8.5, *p* = 0.01) and abduction scores (F 8.3, *p* = 0.011) in the PRP and bursal stem cell therapy group in the second postoperative month of follow-up.

The effect of PRP and bursal stem cell therapy remained constant during late follow-up, from the fourth to sixth postoperative month, with the postoperative CMS regarding pain remaining statistically significantly higher in the stem cell therapy group (F 4.8, *p* = 0.008), alongside reduced night-time pain (F 7.4, *p* = 0.015), improved recreation ability (F 4.8, *p* = 0.044) and reduced activity restriction (F 5.8, *p* = 0.028), as shown in Table 2.

**Figure 7:**
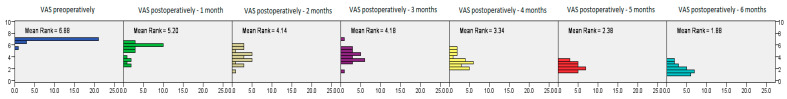
Postoperative VAS score showing significantly improved results in patients treated with PRP and bursal stem cell therapy measured at different time intervals, from the preoperative check-up to the 6-month check-up (Friedman’s test, χ^2^ = 109.199, df = 6, *p* < 0.0001).

Next in the analysis, Friedman’s test was used to show the changes between each follow-up time interval in the PRP and bursal stem cell group. When measuring the changes in these variables at all seven time points, Friedman’s test showed that the levels of preoperative and postoperative pain differed significantly, with a clear drop in VAS pain scores in the second postoperative month, as indicated by the general linear mixed-effects model earlier (Friedman’s test, χ^2^ = 109.199, df = 6, *p* < 0.0001, Figure 7).

**Figure 8:**
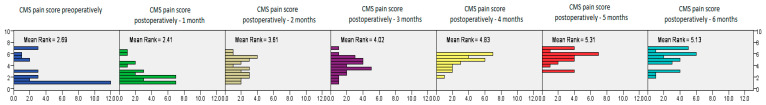
Postoperative CMS pain scores showing significantly improved results in patients treated with PRP and bursal stem cell therapy measured at different time intervals, from the preoperative check-up to the 6-month check-up (Friedman’s test, χ^2^ = 53.950.199, df = 6, *p* < 0.0001).

When analyzing CMS values related to postoperative pain, the same relationship is evident, with the postoperative functional CMS increasing significantly by the second measurement interval in the second postoperative month (Friedman’s test, χ^2^ = 53.950.199, df = 6, *p* < 0.0001, Figure 8).

**Figure 9:**
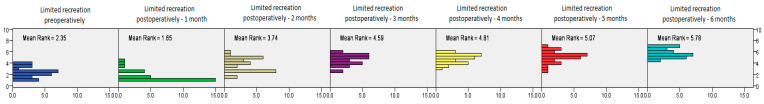
The postoperative CMS for limitations in recreation measured at different time intervals, showing significantly improved results in patients treated with PRP and bursal stem cell therapy, from the preoperative check-up to the 6-month check-up (Friedman’s test, χ^2^ = 108.705, df = 6, *p* < 0.0001).

After analyzing CMS values related to postoperative recreation limitations, the test confirmed a significant improvement at all postoperative time intervals, with the postoperative CMS increasing significantly by the third measurement interval and then continuously increasing in all the later postoperative months (Friedman’s test, χ^2^ = 108.705, df = 6, *p* < 0.0001, Figure 9).

**Figure 10:**
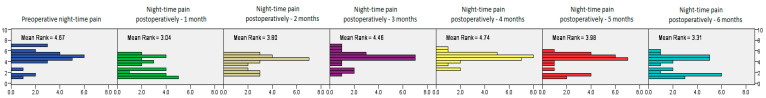
The postoperative CMS for night-time pain measured at different time intervals, showing significantly improved results in patients treated with PRP and bursal stem cell therapy, from the preoperative check-up to the 6-month check-up (Friedman’s test, χ^2^ = 108.705, df = 6, *p* < 0.0001).

Finally, after analyzing CMS values related to postoperative night-time pain, the test confirmed a significant improvement at all postoperative time intervals, with the postoperative CMS increasing significantly by the third measurement interval and then continuously increasing in all the later postoperative months (Friedman’s test, χ^2^ = 108.705, df = 6, *p* < 0.0001, Figure 10).

## 4. Discussion

Our study showed that patients receiving the additional combined PRP and bursal stem cell therapy alongside arthroscopic surgery had a statistically significant decrease in postoperative pain and an increase in functionality compared to patients undergoing arthroscopic surgery alone. Postoperative pain improvements were observed both in the short term, noted in the second postoperative month, and in the long term, in the third and fifth postoperative months. Improvements in both recreational activity and night-time pain were observed at later stages, in the fifth postoperative month. These findings thus validate our initial hypothesis that incorporating stem cell therapy in shoulder arthroscopic surgery provides significant benefits. Our findings support current papers in the literature, with increasing evidence pointing to the high regenerative potential of stem cells. A crucial point in recovery after surgical repair is tendon healing [4,8,11]. Tendon healing occurs in three overlapping stages: inflammatory, within the first week, fibroblastic, starting 48 h after repair and lasting for 8 weeks, and, finally, remodeling. The inflammatory stage is characterized by the deposition of fibrin and fibronectin by platelets, and the fibroblastic stage is characterized by the formation of type III collagen, which is replaced with type I in the remodeling stage [12]. Due to the hypovascularity and degenerative changes in tendons, there is not adequate restoration of normal histology, leading to improper healing [8,13]. These limited satisfactory outcomes have led to an increase in the number of papers studying the healing potential of PRP, MSCs and growth factors. PRP has been described as promising in the literature, with a study by Shames et al. and others proposing injections of PRP over standard corticosteroid injections due to the effect of platelets shown in healing with the induction of the healing cascade with necessary humeral and cellular mediators [14,15,16,17,18,19]. Hence, in order to further promote healing, the patients in our study received additional PRP injections.

The subacromial bursa is an easily available tissue in shoulder arthroscopy. Almost any rotator cuff surgery starts with a bursectomy in order to better visualize the tear. The subacromial bursa is rich with mesenchymal stem cells, which enable and accelerate tissue regeneration and possess a high capacity for self-renewal, as well as exceptional potential for differentiation into different tissue types. Previous research has pointed to the fact that, due to all their characteristics, mesenchymal stem cells play an essential role in all stages of development of the human body and during the healing process. It has been demonstrated that the natural intrinsic healing mechanism of the rotator cuff starts from the bursal-side epitenon [20]. Other researchers demonstrated in an experimental rat model the migration of autogenous host stem cells in tendon grafts, mainly from the bursal side of the tendon stumps [21]. Furthermore, the bursa, like the peritoneum, possesses the ability to rapidly form protective adhesions, confine inflammation, and enable fibrovascular tissue to migrate and become incorporated (absorb) into a tear, leading to the restoration of the function of the whole tendon or part of it, enhancing the mobility of affected tissue [22]. When comparing the proliferation of bursal stem cells with that of bone marrow cells, a significantly superior capacity for mesenchymal differentiation, including chondrogenesis, osteogenesis and adipogenesis, was seen in the differentiation of bursal stem cells [22]. Research on MSCs dates back to the 1970s [12], with growing interest in recent years due to the persistently unsatisfactory outcomes of shoulder arthroscopy, particularly in rotator cuff pathology, despite technical advancements. The term MSC was introduced in the literature in 1991 by Caplan, defining them as multipotent cells capable of differentiating into various specialized mesenchymal cell types, including osteoblasts, chondrocytes, adipocytes and tenocytes [13]. MSCs are the most extensively researched cells for tissue regeneration in MSK injuries, known for their ability to replace damaged cells, reduce inflammation, support host cell function through paracrine signaling and enhance vascular ingrowth [8]. A study published by Young-Jin et al. showed this effect by demonstrating improvement in the early remodeling of the tendon when investigating bone healing of the Achilles tendon in rats [14]. Researchers then investigated their regenerative potential specifically in the shoulder joint. A study by Hernigou et al. used concentrated bone marrow-derived MSCs as an adjunct to single-row arthroscopic rotator cuff repair and found an enhanced rate of healing and a reduced number of re-tears in the group receiving adjuvant MSCs [16]. Even though bone marrow-derived stem cells have a strong association with improved healing in the shoulder joint, their invasive sampling process highlights the need to explore less invasive and more accessible alternatives. The focus then shifted to the subacromial bursa, an extra-articular tissue located between the acromion and the rotator cuff, easily accessed during surgical intervention for the shoulder joint. It has long been thought of as a source of inflammation and pain, often removed during rotator cuff repair surgeries and subacromial decompressions [7]. A study on rabbits published in 2000 demonstrated the importance of the subacromial bursa and its healing effect [17]. Since then, in vitro studies have shown that human cells derived from the subacromial bursa have mesenchymal properties [9] and meet the minimal criteria for MSCs [7]. Some studies have indicated their superiority in terms of differentiation potential compared to bone-derived MSCs [6]. Further in vivo studies were performed to study this effect in rotator cuff surgery. In a study published by El Safoury et al., the researchers used the subacromial bursa to improve the healing of massive rotator cuff tears by covering the repaired tendon with no harvesting, showing satisfactory results [20]. As mentioned, the subacromial bursa was considered to be the source of inflammation and pain; hence, its use in rotator cuff repair surgeries raised concern about its possible proinflammatory effects postoperatively. This was shown not to be the case, with no clinically relevant negative effects observed when the bursal tissue was harvested and reimplanted in the shoulder joint during rotator cuff repair [21].

PRP, alongside its use in injections alone, can also be added during the harvesting of bursal tissue, as we did in our study. This was also described in the literature in a study by Muench et al., where they additionally used PPP (platelet-poor plasma) and BMAc (bone marrow aspirate concentrate) in combination with PRP, harvested subacromial bursal tissue and demonstrated functional improvements at a minimum one-year follow-up [22]. It is somewhat difficult to compare this directly to our study due to the addition of PPP and BMAc, but it does further emphasize the positive results of these additional techniques in rotator cuff surgeries.

Considering these findings in the literature, the results presented in our study are an extension of the present data, emphasizing the high healing potential of additional stem cell therapy. When assessing all the treatment options for rotator cuff pathology, currently, both conservative and surgical options are being used, with several factors considered in making the decision, such as persistent pain, grade of functional impairment (loss of ROM and decreased strength) and patient-related factors, including age, time elapsed from injury and factors regarding anesthesia. Conservative treatment is thought to be a good option in older patients and those with fewer physical demands [1]. Surgical repair is usually indicated after conservative therapy fails, as shown in a systematic review and meta-analysis by Gurnani and colleagues [3]. The same protocol was followed in our study, where patients underwent surgery after previous failed conservative management (including physiotherapy and/or cortisone injections). Nowadays, arthroscopy is chosen over the open approach, leading to faster recovery. The aim of this surgery is to enable secure tendon fixation until healing occurs, further emphasizing the previously stated importance of healing in overall patient outcomes [2]. Regardless of the development of new surgical techniques and biological augmentation, rehabilitation is still an important factor in the overall improvement in patient quality of life. A systematic review by Littlewood and colleagues investigated early initiation of rehabilitation and patient-directed active exercise but did not find strong evidence on the topic; hence, early active movements are not strongly recommended in the current literature [1]. Therefore, active assisted and active non-assisted ROM exercises started from only from the fifth postoperative week, aiming for full ROM, which is expected approximately after 10–12 weeks. Strengthening exercises were started along with the active ROM exercises, and submaximal strength was expected to be achieved 4–6 months after surgery. A return to shoulder-demanding work or sports is usually possible after 6 months, depending on a patient-specific assessment. It is worth highlighting that patient care continues beyond surgery, and effective rehabilitation protocols are essential for the success of surgical interventions, even when stem cell therapy is included.

Despite the promising findings of this study, several limitations should be acknowledged. Firstly, the small sample size limits the generalizability of the results and reduces the statistical power needed to detect subtle differences between groups. Since the experimental arm received MSC + PRP and the control arm did not, the results cannot distinguish between the effects of MSCs from those of PRP. For the patients’ benefit, we did not split the treatment groups into two additional intervention groups, enabling maximal treatment effect but also adding bias to the outcome analysis. Additionally, the short-term follow-up period may not capture long-term outcomes or potential long-term complications associated with surgery or stem cell therapy use. This study’s non-randomized design introduces the possibility of selection bias, and the lack of blinding could influence subjective assessments of pain and functional improvements. Furthermore, variability in the source, processing and administration of PRP and bursal stem cells could not be completely controlled, which may affect the consistency and reproducibility of the intervention. Future studies with larger, randomized controlled trials and extended follow-up are necessary to validate these preliminary findings and determine the safety and efficacy of combining stem cell therapy with arthroscopic rotator cuff repair.

## 5. Conclusions

This study found that patients who received PRP and bursal stem cell therapy alongside arthroscopic shoulder surgery had significantly lower postoperative pain scores in the second postoperative month, evidenced by an improved CMS for pain, with sustained benefits in the late postoperative period, up to six months. Additionally, by the fifth postoperative month, patients in the stem cell group reported reduced limitations in recreation and night-time pain, suggesting that stem cell therapy enhances early pain relief, functional recovery, and long-term postoperative outcomes.

## Figures and Tables

**Table 1:** Descriptive statistics of variables in the sample.

All participants	N = 27	
Male	N = 12	
Female	N = 15	
Age in years		
Average ± SD	57.04 ± 8.23	
Values according to treatment group		
Age in years	Intervention group 57.06	Control group 57
Laterality	10 right, 7 left	8 right, 2 left
Active in sports	10/17	6/10
Supraspinatus injury	17/17	8/10
Infraspinatus injury	1/17	0/10
Teres minor injury	0/17	0/10
Teres major injury	0/17	0/10
Long bicep injury	13/17	8/10
Subscapularis injury	7/17	4/10
Average width of rupture in centimeters	3.89 ± 1.45	4.3 ± 1.5

**Table 2:** Comprehensive overview of study variables: preoperative and postoperative outcomes after binary logistic regression, with the use of bursal stem cells and PRP as the main difference between the groups. Significant correlations are flagged with an asterisk and bold text.

Study Variables	Values According to Treatment Group	
Preoperative	Intervention Group	Control Group
VAS pain	9.8 ± 2	8.9 ± 2.4
CMS pain score	8.8 ± 3.9	12 ± 4.9
Limitation of activity	1.5 ± 0.5	1.5 ± 0.5
Limitation in recreation	1.8 ± 0.5	1.6 ± 0.6
Night-time pain	1.7 ± 0.6	1.7 ± 0.6
Elevation without pain	2.7 ± 0.8	2.8 ± 0.9
CMS limitation score	3.9 ± 1.5	4.3 ± 1.5
Forward flexion score	6.8 ± 2.1	7.6 ± 2.3
Abduction score	5.9 ± 1.9	6.6 ± 2
External rotation score	5.2 ± 2.4	5.4 ± 2.2
Internal rotation score	5.2 ± 2.5	3.6 ± 2.3
Range of motion CMS	23.1 ± 7.4	23.2 ± 7
Average strength score	4 ± 2.5	3.7 ± 2.6
**Early postoperative follow-up at two to three months**		
VAS pain	2.3 ± 2 *	4.2 ± 2.1 *
CMS pain score	12.8 ± 2.3 *	10.2 ± 3.2 *
Limitation of activity	2.8 ± 1	2.8 ± 1
Limitation in recreation	2.4 ± 1.2	2.6 ± 1.1
Night-time pain	1.4 ± 0.6	1.4 ± 0.6
Elevation without pain	8.4 ± 1.7 *	6.8 ± 2.4 *
CMS limitation score	14.9 ± 3.5	13.6 ± 4.3
Forward flexion score	9.1 ± 1.2 *	7.2 ± 2.6 *
Abduction score	8.5 ± 1.4 *	6.6 ± 2.5 *
External rotation score	7.7 ± 2	7 ± 2.5
Internal rotation score	6.4 ± 2.2	6.2 ± 2.5
Range of motion CMS	31.5 ± 4.1	27 ± 9
**Late postoperative follow-up at four to six months**		
VAS pain	1.1 ± 1.1	0.7 ± 1
Pain-related CMS	14.1 ± 0.9 *	13.2 ± 2.6 *
Limitation of activity	3.7 ± 0.7	3.3 ± 0.9
Limitation in recreation	3.7 ± 0.7 *	3 ± 1. *
Night-time pain	1.7 ± 0.7 *	0.9 ± 0.9 *
Elevation without pain	9.8 ± 3.1	9.8 ± 2.7
CMS limitation score	18.5 ± 2.7 *	17.4 ± 3.8 *
Forward flexion score	10 ± 0	9.8 ± 1.9
Abduction score	10 ± 0	9.8 ± 1.9
External rotation score	9.8 ± 0.9	9.6 ± 1.9
Internal rotation score	9.3 ± 0.9	9.2 ± 1.9
Range of motion CMS	39.1 ± 1.2	38.4 ± 7.3
Average strength score	8.9 ± 2.7	8.9 ± 3.7

## Data Availability

Data related to the manuscript’s results will be made available by the corresponding author upon reasonable request.
